# The Relationship Between Physical Activity and Sarcopenia in the Elderly

**DOI:** 10.7759/cureus.104507

**Published:** 2026-03-01

**Authors:** Fajri Fadhillah Hidayat, Erica Kholinne

**Affiliations:** 1 Surgery, Faculty of Medicine, Universitas Trisakti, Jakarta, IDN; 2 Orthopaedics and Traumatology, Gatam Institute, Eka Hospital, Tangerang, IDN

**Keywords:** elder, indonesia, ipaq-sf, physical activity, sarcopenia

## Abstract

Background

Sarcopenia is a significant health problem in older adults, characterized by a progressive decline in muscle mass and muscle strength, and is associated with an increased risk of falls, disability, and reduced quality of life. Physical activity is known to play an important role in maintaining muscle function; however, evidence regarding the relationship between physical activity level and sarcopenia among older adults remains inconsistent, particularly in Indonesia. This study aimed to determine the relationship between physical activity and possible sarcopenia in older adults.

Methods

This study used an analytical observational design with a cross-sectional approach. A total of 67 older adults aged ≥65 years residing in a social institution in West Jakarta who met the inclusion criteria were enrolled. Physical activity was assessed using the International Physical Activity Questionnaire-Short Form (IPAQ-SF) and classified into light, moderate, and high activity levels. Possible sarcopenia was evaluated according to the Asian Working Group for Sarcopenia (AWGS) criteria, using calf circumference measurement and handgrip strength assessment. Data were analyzed using univariate and bivariate analyses. The association between physical activity and possible sarcopenia was tested using Fisher’s Exact test with a significance level of 0.05.

Results

Most participants had a low level of physical activity (71.6%) and were categorized as having possible sarcopenia (59.7%). Bivariate analysis showed no statistically significant association between physical activity level and possible sarcopenia among older adults (p = 0.421). However, effect size analysis indicated that participants with low physical activity had higher odds of possible sarcopenia compared with those with higher activity levels (OR = 2.03; 95% CI: 0.76-5.46), although this association was not statistically significant.

Conclusion

There was no significant relationship between physical activity and possible sarcopenia in this study. These findings suggest that other factors, such as nutritional status, comorbidities, and individual characteristics, may contribute to the development of possible sarcopenia. Further studies with longitudinal designs and more comprehensive assessment methods are needed to clarify this relationship.

## Introduction

The increase in the older adult population is an unavoidable global phenomenon. The World Health Organization (WHO) estimates that the elderly population will rise significantly over the coming decades [1]. Population aging has led to a substantial increase in the number of individuals aged ≥65 years worldwide, with studies documenting a substantial rise in the age-related disease burden in this age group over recent decades [2]. The aging process is accompanied by various physiological changes, one of which is sarcopenia, a condition characterized by a progressive decline in skeletal muscle mass and strength with advancing age [3]. This decline has been reported to begin as early as the third to fourth decades of life, with individuals losing approximately 3-8% of muscle mass per decade after the age of 30, and the rate of loss accelerating further after the age of 65 [4,5].

Sarcopenia is a multifactorial condition associated with an increased risk of falls, fractures, disability, reduced quality of life, and mortality among older adults [6]. The prevalence of sarcopenia in the elderly population has been reported to range from 5% to 16% and increases with advancing age, particularly among individuals over 80 years old. Due to its significant impact on health and physical function, sarcopenia has become a primary health concern in geriatric populations [7].

Physical activity is one of the key factors involved in maintaining muscle mass and strength. It can enhance muscle protein synthesis, improve mitochondrial function, and reduce chronic inflammation, which plays a role in the pathophysiology of sarcopenia [8,9]. Insufficient physical activity has been identified as a significant risk factor for the development of sarcopenia in older adults [10].

However, current evidence remains inconclusive on whether physical activity reduces the incidence of sarcopenia among older adults [11]. Ko et al. reported that higher levels of physical activity were associated with a lower risk of sarcopenia [12], whereas Wakana et al. found the opposite [13]. Variations in study populations and methodological approaches may explain these inconsistent findings. Therefore, well-designed further studies, especially those involving the Indonesian population, are necessary to provide more unmistakable evidence on the relationship between physical activity and possible sarcopenia in older adults.

## Materials and methods

The research was conducted in November 2025 at Panti Sosial Tresna Werdha Budi Mulia 2, Cengkareng, West Jakarta, Indonesia. This study used an analytical, observational, cross-sectional design. The study population consisted of all older adults aged ≥65 years residing at the social care facility. Sampling was carried out using consecutive non-random sampling. The inclusion criteria were the ability to communicate effectively and the willingness to participate in the study. "Ability to communicate effectively" was defined as the participant's capacity to understand and respond appropriately to standardized interview questions and instructions required for completion of the International Physical Activity Questionnaire-Short Form (IPAQ-SF) and for performance-based assessments [14]. Participants who were unable to provide consistent responses necessary for metabolic equivalent of task (MET) calculations or possible sarcopenia classification according to the Asian Working Group for Sarcopenia (AWGS) criteria [15] were excluded. The exclusion criteria included severe neurological disorders, severe musculoskeletal disorders, use of medications affecting muscle metabolism, and severe cognitive impairment. The Research Ethics Commission of Fakultas Kedokteran Universitas Trisakti approved the study (registration number: 047/KER/FK/09/2025), and all participants were fully informed of the study's procedures and provided written consent.

Physical activity was assessed using the IPAQ-SF, administered through structured face-to-face interviews by trained interviewers to ensure comprehension among older participants. Each participant was guided to recall physical activities performed during the previous seven days, including walking, moderate-intensity activities, and vigorous-intensity activities across daily routines. Interviewers used simple explanations, examples of common activities, and clarification questions to minimize recall bias and improve response accuracy. Total physical activity was calculated in MET-minutes per week according to the standard IPAQ-SF scoring protocol by multiplying frequency (days/week), duration (minutes/day), and assigned MET values for each activity domain. Participants were then classified into low, moderate, and high physical activity levels: low physical activity if they did not meet the criteria for moderate or high categories; moderate physical activity if they achieved at least 600 MET-minutes/week through a combination of walking, moderate-intensity, or vigorous-intensity activities; and high physical activity if they accumulated at least 3,000 MET-minutes/week or engaged in vigorous-intensity activity on ≥3 days totaling ≥1,500 MET-minutes/week. Possible sarcopenia was evaluated according to the Asian Working Group for Sarcopenia (AWGS) 2019 criteria [15].

Measurement of calf circumference

Calf circumference was measured by a trained physician at the time of admission. To reduce the influence of edema, the measurements were performed in the morning after muscle mass assessment. Participants were positioned supine with the knee flexed at approximately 90° and the feet and ankles relaxed. A flexible measuring tape was placed perpendicular to the long axis of the leg, and the largest circumference was recorded to the nearest 0.1 cm. Prior to measurement, the physician also assessed for pitting edema. Possible sarcopenia was identified when the calf circumference was measured below 34 cm in men and below 33 cm in women.

Measurement of handgrip strength

Handgrip strength was assessed in both hands with a hand grip dynamometer (Onemed® EH101, Onemed, Java, Indonesia), and the highest value obtained was used for analysis. The test was conducted in either a standing or seated position, depending on the patient’s ability, with the arms held straight at the sides. Low muscle strength was defined as a handgrip strength of <28 kg in men and <18 kg in women.

Statistical analysis

Analyses were performed using SPSS version 27.0 (IBM Corp., Armonk, NY). Univariate analysis was used to describe respondent characteristics, while bivariate analysis was conducted to assess the association between physical activity and possible sarcopenia. Fisher's exact test was applied with a significance level of <0.05. In addition to significance testing, effect size was estimated by calculating crude odds ratios (ORs) with 95% confidence intervals (CI) to describe the magnitude and direction of the association between physical activity level and possible sarcopenia.

## Results

This study involved 67 elders (35 males and 32 females). The ages ranged from 65 to 81 years. The general characteristics of the respondents are presented in Table 1.

**Table 1:** Demographic characteristics of the participants.

Variable	Frequency (n = 67)	Percentage (%)
Age (years) (Mean ± SD)	70.2 ± 8.1	-
Sex		
Female	35	52.2
Male	32	47.8
Possible sarcopenia		
Yes	40	59.7
No	27	40.3
Physical activity		
Light activity	48	71.6
Moderate activity	17	25.4
High activity	2	3.0

The results of data analysis showed that the gender distribution of respondents was relatively balanced, with 32 male respondents (47.8%) and 35 female respondents (52.2%). Regarding educational level, the majority of respondents had no formal education, accounting for 22 individuals (32.8%). The smallest proportion was respondents who had completed higher education, totaling eight individuals (11.9%). The remaining respondents had completed elementary school (15 individuals; 22.4%), junior high school (10 individuals; 14.9%), and senior high school (12 individuals; 17.9%).

Based on physical assessments using calf circumference and handgrip dynamometer measurements, most respondents were classified as having possible sarcopenia, with 40 participants (59.7%). In contrast, 27 respondents (40.3%) were not categorized as having possible sarcopenia. Furthermore, most respondents had a low level of physical activity, totaling 48 individuals (71.6%). Respondents with moderate physical activity numbered 17 individuals (25.4%), while those with high physical activity constituted the smallest group, with only two individuals (3.0%).

In the bivariate analysis assessing the relationship between physical activity and possible sarcopenia among older adults (Table 2), the chi-square test could not be applied because its assumptions were not met. Fisher’s exact test demonstrated no statistically significant association between physical activity level and possible sarcopenia (p = 0.421). Nevertheless, a descriptive trend was observed: possible sarcopenia was more prevalent among respondents with light physical activity (64.6%) than among those engaging in moderate (47.1%) or high physical activity (50%). This pattern suggests a tendency toward lower prevalence of sarcopenia with increasing physical activity levels. Effect size analysis indicated that participants with low physical activity had higher odds of possible sarcopenia compared with those with higher activity levels (OR = 2.03; 95% CI: 0.76-5.46). However, this association was not statistically significant, and the confidence interval was wide, reflecting limited statistical power.

**Table 2:** The relationship between physical activity and sarcopenia among older adults. IPAQ: International Physical Activity Questionnaire.

IPAQ score	Possible sarcopenia, n (%)	No possible sarcopenia, n (%)	Total	p-value
Low	31 (64.6)	17 (35.4)	48	0.421
Moderate	8 (47.1)	9 (52.9)	17	
High	1 (50)	1 (50)	2	

## Discussion

This finding is consistent with the inclusion criteria, which were specifically designed to focus on the older adult population. Age is a critical factor in the development of sarcopenia, as degenerative processes begin to increase significantly after age 60, leading to reductions in muscle mass, strength, and tissue quality [16]. The gender distribution of respondents was relatively balanced, with 35 women (52.2%) and 32 men (47.8%). This balance is advantageous for the study as it reduces potential gender-related bias. However, epidemiologically, several studies have reported that postmenopausal women are at a higher risk of developing sarcopenia due to decreased estrogen levels, which affect muscle metabolism [17]. Conversely, other studies have indicated that men are also vulnerable to greater age-related muscle mass loss as a result of declining testosterone levels [18]. Although the study population shared relatively similar living conditions, the lack of multivariable adjustment means residual confounding cannot be ruled out.

In this study, the proportion of possible sarcopenia was 40 participants (59.7%), with 27 individuals (40.3%) classified as not having possible sarcopenia. This prevalence is relatively high compared with several studies conducted among older populations in Asia. For instance, a study by Chew et al. in Singapore reported a sarcopenia prevalence of 23-46%. In comparison, it ranges from 18% to 41% among Asian community-dwelling older adults. The high prevalence observed in this study may be influenced by the characteristics of the respondents, most of whom had low levels of education and predominantly engaged in low-intensity physical activity [19].

Regarding physical activity, most respondents were categorized as having light activity (71.6%), followed by moderate (25.4%) and high (3.0%). The predominance of low-intensity physical activity among older adults is a typical pattern reported across studies, particularly in elderly populations in developing countries. Reduced physical activity in older adults may be influenced by limited mobility, comorbidities, low health literacy, and the lack of a supportive environment for physical activity [20]. Low physical activity is recognized as an essential risk factor for sarcopenia. A cohort study by Steffl et al. reported that older adults with low physical activity levels had a two to three times higher risk of developing sarcopenia compared with those who remained physically active [11].

The findings of this study indicate that there was no significant association between physical activity and possible sarcopenia among older adults (p > 0.05). This result is consistent with the study by Wakana et al., which demonstrated that although physical activity influences muscle function, its direct association with sarcopenia is not always significant after adjustment for age, nutritional status, and chronic disease [13]. The authors emphasized that multifactorial factors, such as malnutrition, inflammation, and chronic diseases, may act as confounders, weakening the relationship between physical activity and sarcopenia [13]. Similarly, a study by Seo et al. found that moderate-to-high-intensity physical activity had the most significant effect on maintaining muscle mass. In contrast, low-intensity physical activity did not provide a meaningful protective effect against sarcopenia. They also found that gender differences influenced the physiological response to physical activity [21].

The non-significant findings of this study differ from several previous studies that have demonstrated an essential role of physical activity in preventing sarcopenia. For example, a meta-analysis by Steffl et al. reported that older adults with low physical activity levels had a two to three times higher risk of sarcopenia compared with physically active older adults [11]. Similarly, a study by Tabata et al. showed that regular physical activity, particularly resistance training and moderate-intensity exercise, was associated with increased muscle mass and strength in older adults [22].

However, the results of the present study are inconsistent with those reported by Ko et al., who demonstrated that physical activity, particularly moderate-intensity aerobic exercise and resistance training, was significantly associated with a lower risk of sarcopenia in older adults. Their findings highlighted that regular physical activity contributes to improvements in muscle mass, strength, and physical performance, which are fundamental components in the diagnosis of sarcopenia [12]. In addition, a study by Sato et al. found that high sedentary time and low physical activity were strong risk factors for sarcopenia, especially among older adults with metabolic comorbidities. They also highlighted that the protective effects of physical activity become evident when both duration and intensity meet the WHO's minimum recommendations [23].

Several factors may explain the differences between this study's findings and those of previous research. First, not all forms of physical activity measured by the IPAQ provide sufficient stimulus to increase or maintain muscle mass. Low-intensity activities, such as leisurely walking, may not be adequate to prevent age-related muscle loss. This is consistent with the findings of Marzetti et al., who reported that only moderate-to-high-intensity physical activity and muscle-strengthening exercises have a significant impact on slowing the progression of sarcopenia [24]. Second, most respondents in this study had a low level of education, which may influence their understanding of healthy lifestyle behaviors, including the quality and appropriate duration of physical activity. A survey by Melsæter et al. found that educational level was significantly associated with physical activity and functional performance among older adults, suggesting that education may act as a confounding factor [25]. Third, other lifestyle-related factors, such as nutritional status, comorbidities, and hormonal status, which were not analyzed in this study, may influence the development of sarcopenia despite physical activity. According to Cruz-Jentoft et al., sarcopenia is a multifactorial condition, and interactions among multiple risk factors may attenuate the protective effects of physical activity [6].

This study has several limitations. First, the cross-sectional design does not allow the determination of causal relationships among the variables examined. Because exposure and outcome were measured simultaneously, it is not possible to determine whether low physical activity increased the risk of possible sarcopenia or whether individuals with sarcopenia were less physically active due to reduced muscle strength and functional capacity. Therefore, reverse causation is highly plausible, and the observed association should not be interpreted as directional or causal. Second, the assessment of physical activity using the IPAQ relies on self-reported recall among older adults, which may introduce recall bias and measurement error. Third, the relatively small sample size, together with the unequal distribution of physical activity levels, particularly the minimal number of participants in the high physical activity category, may have limited the statistical power to detect a true association. Moreover, the IPAQ does not specify whether reported activities include structured strength training, an important component of sarcopenia prevention. In addition, the IPAQ was originally developed and validated primarily in adults aged 15-69 years, and its accuracy in frail or institutionalized older adults is limited. Multivariable regression analysis was not performed to adjust for potential confounders, including age, sex, BMI, nutritional status, and comorbid conditions. Therefore, unmeasured or uncontrolled confounding may have influenced the results.

Furthermore, the assessment of sarcopenia was limited to calf circumference and handgrip strength, which serve as screening components and do not constitute a full diagnostic assessment that includes muscle mass and physical performance; therefore, the classification reflects possible sarcopenia rather than confirmed sarcopenia. The study was conducted in a single social care facility, limiting the generalizability of the findings to other older adult populations. In addition, the participants were exclusively institutionalized older adults, who may share relatively similar living conditions, daily routines, access to healthcare, and socio-economic characteristics. These factors may differ from those in community-dwelling older adults and could influence physical activity patterns and the risk of sarcopenia. As a result, the findings may not be fully generalizable to the broader elderly population, particularly those from diverse socio-economic backgrounds. Therefore, the absence of a statistically significant relationship should be interpreted cautiously, as it may reflect sample size constraints, imbalance in exposure categories, and measurement limitations rather than the absence of a true association.

## Conclusions

In this study, a high proportion of older adults living in a social care facility were classified as having possible sarcopenia based on the AWGS screening criteria, and most participants reported low levels of physical activity. In this cross-sectional sample, no statistically significant association was detected between physical activity level and possible sarcopenia using Fisher’s exact test. These findings should be interpreted cautiously in light of methodological constraints, including imbalance across physical activity categories, absence of adjustment for potential confounders, and reliance on screening-based sarcopenia classification, which may have introduced outcome misclassification. Additionally, the institutionalized study population limits generalizability to community-dwelling older adults. Rather than indicating the absence of a relationship, the results highlight the complexity and multifactorial nature of sarcopenia. Future studies employing longitudinal designs, confounder-adjusted analyses, and comprehensive diagnostic criteria are needed to better clarify the role of physical activity in sarcopenia among older adults.
